# Low-Level Zoonotic Transmission of Clade C MERS-CoV in Africa: Insights from Scoping Review and Cohort Studies in Hospital and Community Settings

**DOI:** 10.3390/v17010125

**Published:** 2025-01-17

**Authors:** Andrew Karani, Cynthia Ombok, Silvia Situma, Robert Breiman, Marianne Mureithi, Walter Jaoko, M. Kariuki Njenga, Isaac Ngere

**Affiliations:** 1 Global Health Program, Washington State University Global Health-Kenya, Nairobi 00200, Kenya; andrew.karani@wsu.edu (A.K.); cynthia.ombok@wsu.edu (C.O.); silvia.situma@wsu.edu (S.S.); mkariuki.njenga@wsu.edu (M.K.N.); 2 Department of Medical Microbiology, University of Nairobi, Nairobi 00200, Kenya; marianne@uonbi.ac.ke (M.M.); wjaoko@kaviuon.org (W.J.); 3 Rollins School of Public Health, Emory University, Atlanta, GA 30322, USA; rbreima@emory.edu; 4 Paul G Allen School of Global Health, Washington State University, Pullman, WA 98165, USA

**Keywords:** MERS-CoV, zoonotic, clades, epidemiology, camel, infections, serology, prevalence, transmission, symptoms

## Abstract

Human outbreaks of Middle East respiratory syndrome coronavirus (MERS-CoV) are more common in Middle Eastern and Asian human populations, associated with clades A and B. In Africa, where clade C is dominant in camels, human cases are minimal. We reviewed 16 studies (n = 6198) published across seven African countries between 2012 and 2024 to assess human MERS-CoV cases. We also analyzed data from four cohort studies conducted in camel-keeping communities between 2018 and 2024 involving camel keepers, camel slaughterhouse workers, and hospital patients with acute respiratory illness (ARI). The analysis showed a pooled MERS-CoV prevalence of 2.4% (IQR: 0.6, 11.4) from 16 publications and 1.14% from 4 cohort studies (n = 2353). Symptomatic cases were rarely reported, with most individuals reporting camel contact, and only 12% had travel history to the Middle East. There was one travel-associated reported death, resulting in a mortality rate of 0.013%. The findings suggest a low camel-to-human transmission of clade C MERS-CoV in Africa. Ongoing research focuses on genomic comparisons between clade C and the more virulent clades A and B, alongside the surveillance of viral evolution. This study highlights the need for continuous monitoring but indicates that MERS-CoV clade C currently poses a minimal public health threat in Africa.

## 1. Introduction

Over the last 25 years, the zoonotic transmission of newly emergent coronaviruses to humans has resulted in severe multi-continent epidemics of respiratory diseases, including the severe acute respiratory syndrome (SARS) first detected in 2002 in China caused by SARS coronavirus (SARS-CoV), Middle East respiratory syndrome (MERS) detected in 2012 in Saudi Arabia caused by MERS coronavirus (MERS-CoV), and the COVID-19 pandemic detected in 2019 in China caused by SARS-CoV-2 [1,2,3]. Of these viruses, only MERS-CoV has a domestic animal reservoir: dromedary camels, which transmit the virus to humans in close contact through aerosol droplets [4,5].

There are three genetically distinct clades of MERS-CoV, A, B, and C, with clades A and B continuing to spread through the Middle East, Asia, and other countries through primary camel contact and secondary human–human transmission fueled by close contact in intensive care units and travel, resulting in 2613 confirmed human cases and a case fatality rate of 36% as recorded at the time of the last reported case in April 2024 [6,7]. Over 80% of these cases were reported from Saudi Arabia, 55% being infected through primary camel contact and the rest through secondary human-to-human transmission [8]. Most (>80%) clade A/B clinical cases occur in older persons (median age = 58 years), having at least one underlying medical condition such as chronic renal failure, heart failure, diabetes, and/or hypertension [9]. Clade A MERS-CoV caused the initial wave of cases but was quickly replaced by clade B as the primary circulating clade in the Middle East beyond the initial outbreak [10,11,12].

Despite being home to >80% of the global dromedary camel population and being the source market for most camels sold in the Middle East, Africa has never reported a human MERS-CoV outbreak associated with primary camel exposure [13]. Most studies indicate that only clade C virus circulates in the continent, associated with documented outbreaks in camels and >70% seroprevalence in most camel populations, but limited serologic evidence of human exposure or symptomatic infection has been reported in humans [14,15,16]. The paucity of human cases in Africa may be explained by viral plasticity, resulting in the inefficient transmission and/or weakened virulence of clade C, as supported by in vitro and ex vivo studies [17]. Alternatively, this may be due to poor disease surveillance and reporting among the marginalized nomadic pastoralist populations that inhabit remote arid lands where camels are reared [18]. Here, we combined our intensive hospital- and community-based MERS-CoV studies in northern Kenya with a scoping review of studies across Africa to assess the levels of camel-to-human virus transmission and morbidity in humans.

## 2. Materials and Methods

### 2.1. Scoping Review

We conducted a scoping review to identify and evaluate reported human MERS-CoV cases across Africa using Preferred Reporting Items for Systematic Reviews and Meta-analyses (PRISMA) guidance and registered the protocol on Open science Framework. A comprehensive literature search using the PubMed, Research4life, Elsevier, Google Scholar, Cochrane, EMBASE, and HINARI databases was conducted to identify peer-reviewed publications on human MERS-CoV infections in Africa between January 2012 and September 2024. Search terms were informed by keywords using the Population, Intervention, Control group, Outcome (PICO) guidance to ensure the broadest results (Supplementary Table S1).

### 2.2. Selection of Articles

The selected articles were independently screened by two researchers for inclusion and exclusion. Studies with human cases/human samples of MERS-CoV from African countries; epidemiological research; molecular epidemiology studies; cohort studies; case–control studies; cross-sectional studies and mixed method studies; reviews (scoping, systematic); and clinical research including clinical studies, diagnostic studies, prognostic studies, and case reports/series studies were all included in this scoping review. Basic MERS-CoV research studies, laboratory studies and cellular studies, biochemistry studies, genetic/genomic studies, commentaries, editorials, opinion pieces, perspectives, dissertations (unless published in a peer review), non-English translated studies, and MERS-CoV studies conducted outside Africa within the same period were excluded. Data from selected articles were manually recorded in Microsoft Excel using the following variables: Article identification, including the Source of the article, Year of publication, Full title, Author, Reason for inclusion, and Country of origin. The key outcomes of interest were prevalence, morbidity, and mortality. Other variables included the type of sample, laboratory test used, occupation, median age, sex, study design, postulated origin/source of infection, and viral mutation.

### 2.3. Empirical Studies in Northern Kenya, 2018–2024

**Figure 1:**
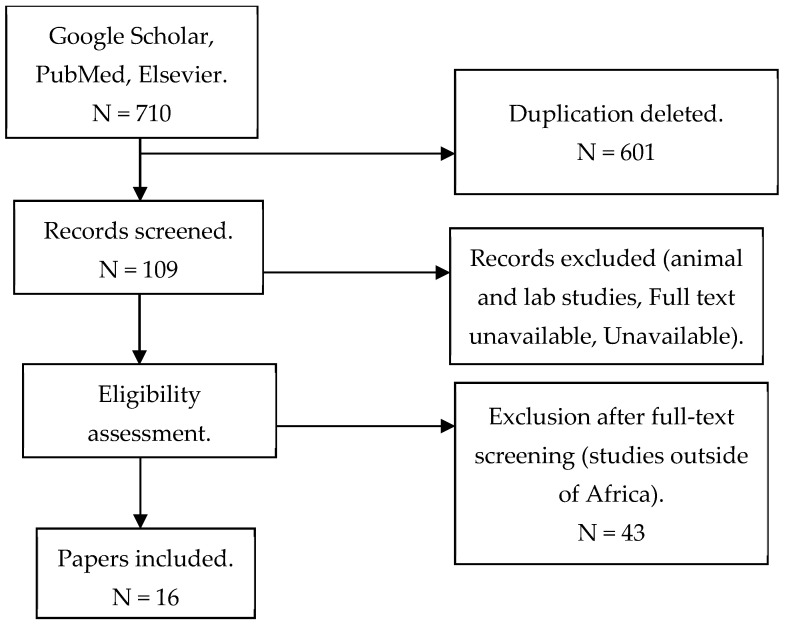
A flowchart outlining the PRISMA process of identifying, screening, and selecting studies.

**Figure 2:**
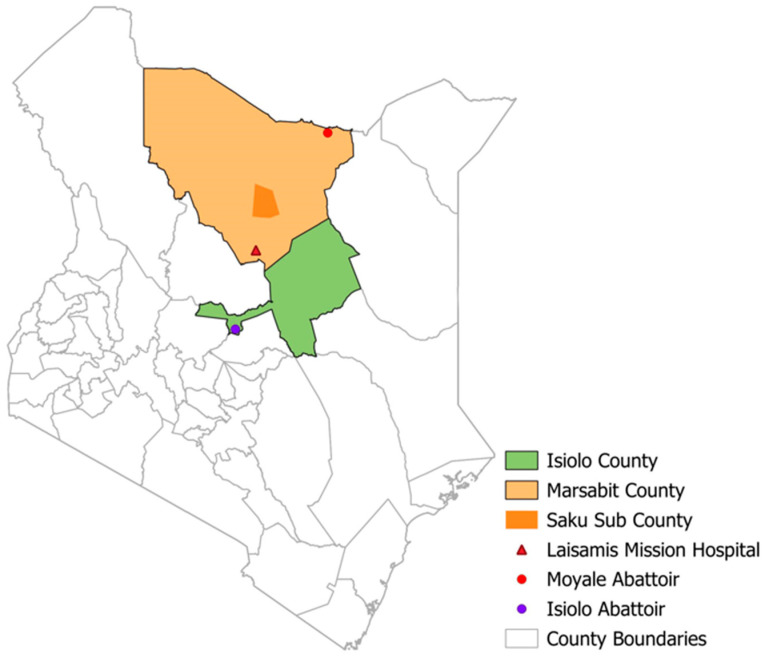
Map of Kenya showing human MERS-CoV study sites.

We analyzed data from four ongoing studies on human MERS-CoV among camel pastoralist communities in Isiolo and Marsabit counties in northern Kenya between 2018 and 2024 (Figure 1 and Figure 2). These included two community cohort studies enrolling 351 camel herders, a 2-year prospective cohort of slaughterhouse workers (n = 124), and two hospital cross-sectional surveillance studies enrolling patients presenting with flu-like symptoms at Marsabit County Referral Hospital (n = 935) and Laisamis Catholic Mission Hospital (n = 942). We used structured questionnaires to collect data on risk factors, clinical symptoms, and comorbidities.

### 2.4. Sample Collection and Laboratory Testing

Upon consenting, participants provided nasopharyngeal/oropharyngeal (NP/OP) swabs and serum samples. Serum was assessed for IgG antibodies against MERS-CoV using the Euroimmun anti-MERS-CoV enzyme-linked immunosorbent assay test kit (EUROIMMUN Medizinische Labordiagnostika AG, Lubeck, Germany) according to the manufacturer’s instructions. This kit was used to semi-quantitatively detect IgG antibodies against MERS-CoV in serum by binding antibodies to viral antigens in precoated wells, followed by a colorimetric reaction indicating antibody presence. The results were compared to controls, with a positive result suggesting prior exposure/infection. MERS-CoV ELISA-positive samples underwent a microneutralization assay [19], where positive sera were diluted and mixed with live MERS-CoV or a pseudo-virus, then incubated to allow virus-specific antibodies in the serum to bind. The mixture was then added to a culture of susceptible cells; if neutralizing antibodies were present, they blocked viral infection, indicating prior exposure to MERS-CoV.

To detect MERS-CoV RNA, NP/OP swabs were tested using RT-PCR as previously described [14,15]. Briefly, total nucleic acids were extracted from 200 µL of the sample, followed by a standard RT-PCR test targeting two pre-determined targets. A sample was considered positive if all PCR targets were positive (defined by a Cycle Threshold/CT value < 40).

### 2.5. Statistical Analysis

Data were analyzed using R (version 4.2.0) [20]. The findings of this scoping review were summarized using frequencies and proportions, with continuous variables shown as medians and interquartile ranges (IQRs). Key data included study location, sex, age groups, occupation, travel history, and camel contact. Categorical variables were summarized with frequencies and percentages, while continuous variables (e.g., age) used medians and IQR. Clinical and lab data were combined with demographic information and are presented in tables. Pooled prevalence was used to estimate overall prevalence, weighted by sample size in the scoping review.

## 3. Results

### 3.1. Findings from Scoping Review

Of the 109 articles reviewed, 16 articles [13,14,15,16,19,21,22,23,24,25,26,27,28,29,30] covering seven African countries in East (Kenya, Sudan), North (Egypt, Tunisia, Morocco), and West (Ghana and Nigeria) Africa were included in the analysis (Table 1). The studies covered the period between January 2012 and September 2024. A total of 87.5% (n = 14) of the eligible studies enrolled both human and camel participants. Camel studies were only included in this review if the studies also reported on human testing. The 16 studies included 6198 human participants (median = 262, IQR: 75, 554), most (62.5%) of whom were male with a median age of 42 (IQR: 18, 65). Participant occupations included camel herders (38.0%), abattoir workers (31.0%), and camel farmers (19.0%). The eligible studies also reported the results of 7194 camels, evaluated alongside the human participants. Study types included cross-section serosurveys (n = 9), five longitudinal cohorts (n = 5), one case–control (n = 1), and one case report (n = 1). Most of the studies (62.4%) used a combination of ELISAs and microneutralization assays (MNAs) for serologic confirmation, while 24.0% attempted virus detection by using a polymerase chain reaction (PCR).

### 3.2. Human Prevalence, Morbidity, and Mortality

Of 6198 participants, the median human MERS-CoV pooled prevalence was 2.4% (IQR: 0.6, 11.4). A total of 81.0% of the studies associated the source of human infections with dromedary camels, whereas 12.4% (n = 3) attributed infections to travel from the Middle East. A cohort study conducted in Kenya confirmed three acute but asymptomatic MERS-CoV cases by PCR [14]. While the majority (n = 13) of the studies did not document any clinical symptoms of the participants, three studies reported clinical symptoms including cough (18.8%), fever (12.5%), sore throat (12.5%), difficulty breathing (6.3%), and running nose (6.3%). One mortality was reported from a case study in Tunisia of a 66-year-old male with diabetes, who had traveled back from Qatar and Saudi Arabia prior to the onset of symptoms [23].

### 3.3. Findings from Empirical Studies in Kenya, 2018–2024

Between 2018 and 2024, we conducted four different studies on MERS-CoV in the northern Kenya region, including a household-based camel–human cohort, a longitudinal follow-up of camel slaughterhouse workers, and two hospital-based studies, as shown in Table 2. Some findings from one study ([14,15]) are included in this scoping review. We enrolled a total of 2352 human participants, all associated with the pastoral livelihood of inhabitants of this arid region, 54.1% of them male, and 34.3% below the age of 10 years. Observed underlying conditions like respiratory illnesses were reported by 78.0% of the participants, and visceral leishmaniasis (Kalazar), which is endemic in this region, was reported by 1.7% of the participants (Table 2).

Over half (50.4%) of the participants reported camel contact, as per Table 3. Below is a summary of some of the camel contacts recorded in our studies, including consuming raw products, feeding/herding, milking, and the cleaning of barns (Table 3).

### 3.4. MERS-CoV Prevalence, Morbidity, and Mortality in Human Participants

Of the 2352 participants followed up with in the four studies, 27 (pooled prevalence = 1.14%) were positive for MERS-CoV by either PCR or serology (ELISA + MNA). In the community cohort study, 351 participants were followed up with and tested bimonthly for 1 year, while in the slaughterhouse study, 124 participants were followed up with and tested bimonthly for 2 years. These test results are summarized in Table 4, showing that of 4222 samples tested by PCR, 3 (0.07%) were positive for viral RNA, while 24 (0.6%) were positive for serology.

In the slaughterhouse study, 28.1% of 124 participants developed clinical symptoms over the follow-up period, whereas in the community cohort study, 11.7% of 351 participants did. When we evaluated the prevalence of respiratory symptoms across the four studies, cough (76.7%), running nose (45.6%), and chest pains (28.1%) were the most common (Table 5). Cough had a longer duration (median: 4 days), while other symptoms like sneezing and nasal discharge had shorter (median: 2–3 days) durations. No mortality was reported among participants over the duration of the studies.

## 4. Discussion

This dual-method approach evaluated 8550 human participants at risk of MERS-CoV infection due to either regular contact with camels or travel to the Middle East and confirmed low prevalence and morbidity, and so far, no mortality is associated with autochthonous MERS-CoV clade C transmission in Africa. The overall disease prevalence in the participants, most of whom had camel contact (50–80%), was 2.1%, all of it confirmed via serologic evidence except three PCR-confirmed cases. Clinical respiratory disease was reported in 28% of our study participants and 18.8% of participants in published studies [22,23,24]. However, no clade C-associated mortality was reported. In terms of the one fatality case reported, a 66-year-old male with diabetes from Tunisia who had traveled to the Middle East, thus most likely associated with clade A or B [23].

Our prospective hospital- and community-based studies incorporating 2352 human participants with high-risk occupations ruled out the possibility of a weak surveillance system as the reason for the paucity of human cases associated with clade C virus. In addition, the arid/semi-arid ecosystem of northern Kenya where we conducted our studies mirrors that of the Middle East where clades A and B have caused more severe morbidity and mortality [31]. Therefore, these findings point to a less transmissible and weakly virulent clade C virus as the likely reason for the low disease prevalence and morbidity. This possibility is supported by our three PCR-positive cases from our 2018 cohort of camel herders which was part of our empirical data set. When we sequenced the n = 3 MERS-CoV PCR-positive samples, we obtained three near-complete genomes of MERS-CoV (GenBank PQ538787-9). A comparison of three different positive camel samples from the same cohort showed that all belonged to clade C MERS-CoV and shared >99.9% nucleotide identity to each other and ~99.8% nucleotide identity to previously reported camel MERS-CoV from Kenya and Ethiopia [14]. Furthermore, several studies have also demonstrated that clades A and B are associated with higher virus replication rates and more severe pathologies in ex vivo human lung and bronchus tissues and high replication in vitro compared to clade C [10,32].

To continue analyzing these findings, our team is currently undertaking further functional genomic studies across the three MERS-CoV clades to elucidate possible mechanisms associated with differential virulence among them. In addition, being aware that severe disease associated with clades A and B in recent outbreaks in the Middle East is more frequent and severe among persons with underlying medical conditions [9,33], we will continue conducting longitudinal community cohort studies with broadened enrollment eligibility in northern Kenya regions where we have identified transmission hotspots to further investigate this.

There are several concerns arising from the finding that clade C is less transmissible and less virulent. First, it is evident that clade C virus will continue undergoing functional mutations that enhance its transmissibility and even virulence [34], emphasizing the need for the continuous genomic surveillance of the virus in African camels. Second, clade A or B may be introduced into the continent through human travel or camel sporting activities, resulting in one of these highly virulent strains establishing itself to begin causing severe human outbreaks with high morbidity and mortality [35]. Lastly, the World Health Organization listing MERS-CoV among viruses likely to cause pandemics and pushing the Coalition for Epidemic Preparedness Innovations (CEPI) to pursue its vaccine development is commendable [36,37] and emphasizes the need for ongoing MERS-CoV genomic surveillance in Africa.

Some of the limitations of this analysis include the possibility that some infections that occurred between follow-up visits in the cohort studies were missed due to irregular swabbing. Furthermore, not all household members were enrolled in community studies, underscoring the challenges with virus detection in longitudinal studies. Also, the empirical studies included in this analysis are from one region; however, this region also represents a critical node in the camel export route with three-quarters of the camels being exported to the Middle East origination along this path. Lastly, the serologic assessment of antibodies and MERS-CoV T-cell responses might not have detected mild and asymptomatic MERS-CoV cases, as has been shown in other studies that have shown a lower yield with MERS-CoV and SARS-CoV-2 serologic testing [38,39], probably because of the stringent cut-offs established for infection despite evidence pointing out that clade C infections are mostly subclinical [30]. Despite this limitation, this analysis represents one of the large analyses that has been undertaken to investigate clade C MERS-CoV infection in Africa and forms a basis for future considerations for MERS-CoV surveillance in Africa.

## 5. Conclusions

Our study confirms that currently circulating clade C MERS-CoV strains have limited public health threats, associated with low prevalence and morbidity, and, so far, no mortality. However, given the high circulation in dromedary camels, there is a need for continued surveillance including genomic surveillance in this region to understand the public health threat of clade C MERS-CoV.

## Figures and Tables

**Table 1:** Descriptive summary of publications included in this scoping review.

Country/Region of Origin	No. of Articles	East Africa (Kenya and Sudan)	West Africa (Ghana and Nigeria)	North Africa (Morocco, Tunisia, and Egypt)
**No. of Articles**	16	7	4	5
Sample Size N (Median, IQR *)	262 (75, 554)	261 (178, 623)	575 (254, 932)	28 (24, 179)
**Pooled MERS-CoV Prevalence (Median, IQR)**	2.4 (0.6, 11.4)	1.4 (1.1, 2.25)	12 (6, 21)	8.6 (0, 20)
**Postulated Origin of Human Infection**				
Camel	13 (81.0%)	1	2	4
Environment	1 (6.2%)	0	1	0
Travel	2 (12.4%)	0	1	1
**Human Morbidity**				
Yes	1 (6.2%)	0	0	0
No	13 (81.0%)	7	3	3
**Human Mortality**	1 (6.2%)	0	0	1
**Laboratory Tests**				
ELISA and Neutralization	10 (62.5%)	5	1	4
PCR	5 (31.3%)	2	2	1
T cells	1 (6.2%)	0	1	0

* IQR—interquartile range; ELISA—enzyme-linked immunosorbent assay; PCR—polymerase chain reaction.

**Table 2:** Comparison of demographic characteristics and past medical history of participants enrolled in four studies in northern Kenya, 2018–2024.

Study Type	Community Cohort Study 2018–2021		Hospital Study 2019–2021		Slaughterhouse Study 2022–2024		Hospital Study 2022–2024		All Studies	
	(n = 351)		(n = 935)		(n = 124)		(n = 942)		(n = 2352)	
Variable	n	%	n	%	n	%	n	%	n	%
**Sex**										
Female	139	39.6	382	40.9	26	21	532	56.5	1079	45.9
Male	212	60.4	553	59.1	98	79	410	43.5	1273	54.1
**Age group (years)**										
<10	85	24.2	409	43.7	-	-	312	33.1	806	34.3
11–24	125	35.6	213	22.8	34	27.4	225	23.9	597	25.4
24–49	106	30.2	190	20.3	74	59.7	270	28.7	640	27.2
>50	35	10	123	13.2	16	12.9	105	11.1	279	11.9
**Occupation**										
Student/child	144	41	466	49.8	-	-	4	0.4	614	26.1
Livestock-related	148	42.2	469	50.2	124	100	1	0.1	742	31.5
Non-livestock-related	59	16.8	3	0.3	-	-	3	0.3	65	2.8
**Underlying conditions**										
Respiratory illness	25	7.1	838	89.6	31	25	941	99.9	1835	78
Kalazar *	-	-	16	1.7	-	-	-	-	16	0.7
Other comorbidities	10	0	33	3.5	0	0	63	6.7	106	4.5
**Camel contact**										
Yes	249	70.9	297	31.8	121	97.6	509	54.0	1176	50.4

Other comorbidities: hypertension, diabetes, liver disease, kidney disease. * Kalazar is endemic.

**Table 3:** Camel contact among enrolled participants in studies in northern Kenya, 2020–2024.

Characteristic	Community Cohort Study		Hospital Study 2019–2021		Slaughterhouse Study		Hospital Study 2022–2023		All Studies	
	(n = 351)		(n = 935)		(n = 124)		(n = 942)		(n = 2352)	
	n	%	n	%	n	%	n	%	n	%
**Type of camel contact**										
Consuming raw products	-	-	261	27.8	-	-	480	51	741	31.5
Feeding/herding	245	69.8	102	10.9	2	1.6	128	13.6	477	20.3
Milking	203	57.8	94	10.1	-	-	67	7.1	364	15.5
Cleaning barns	224	63.8	75	8	25	20.2	38	4	362	15.4
Handling meat/hides/skin/offal	-	-	130	13.9	97	78.2	85	9	312	13.3
Treatment/restraining	97	-	43	4.6	20	16.1	37	3.9	197	8.4
Sports/leisure/grooming	31	8.8	70	7.5	70	-	-	4.2	141	6
Slaughter	32	9.1	14	1.5	14	11.3	18	1.9	78	3.3
Assisting in mating/birthing	-	-	16	1.7	-	-	25	2.7	41	1.7
Transport	-	-	-	-	8	6.5	-	-	8	0.3

**Table 4:** ELISA and PCR results of human MERS studies in Kenya, 2018–2024.

	Nasal/Oral Pharyngeal Results (RT-PCR)					Serum Results (ELISA * + MNT)				
		Negative = 4219		Positive = 3		N = 728	Negative = 704		Positive = 24	
Study Type		n	%	n	%	N	n	%	n	%
Community cohort study		1714	99.8	3	0.2	430	414	96.3	16	3.7
Hospital study 2019–2021		320	100.0	0	0.0	0	0	0	0	0.0
Slaughterhouse cohort study		1243	100.0	0	0.0	0	0	0	0	0.0
Hospital study 2022–2024		942	100.0	0	0.0	298	290	97.3	8	2.7

* ELISA—enzyme-linked immunosorbent assay; PCR—polymerase chain reaction; MNT—microneutralization testing.

**Table 5:** Clinical presentation between the community cohort and hospital surveillance studies.

	Community Cohort Study		Hospital Study 2019–2022		Slaughterhouse Study		Hospital Study 2022–2023		All Studies		
Symptoms	(n = 351)		(n = 935)		(n = 124)		(n = 942)		(n = 2352)		Duration of Symptom in Days
	n	%	n	%	n	%	n	%	n	%	Median [Min, Max]
Cough	10	2.8	835	89.3	22	17.7	938	99.6	1805	76.7	4.00 [0.00, 20.0]
Fever	-	-	22	2.4	2	1.6	687	72.9	711	30.2	3.00 [1.00, 14.0]
Difficulty breathing	-	-	204	21.8	-	-	189	20.1	393	16.7	3.00 [1.00, 14.0]
Nasal congestion/stuffiness	8	2.3	12	1.3	-	-	665	70.6	685	29.1	0.00 [0.00, 14.0]
Runny nose	20	5.7	299	32.0	4	3.2	749	79.5	1072	45.6	3.00 [1.00, 14.0]
Sore throat	2	0.6	20	2.1	4	3.2	331	35.1	357	15.2	3.00 [1.00, 14.0]
Chest pain	1	0.3	282	30.2	1	0.8	378	40.1	662	28.1	5.00 [1.00, 20.0]
Headache	-	-	272	29.1	2	1.6	556	59.0	830	35.3	3.00 [1.00, 14.0]
Fatigue	-	-	-	-	-	-	426	45.2	426	18.1	3.00 [1.00, 14.0]
Nausea	-	-	12	1.3	-	-	202	21.4	214	9.1	2.00 [1.00, 14.0]
Sneezing	-	-	3	0.3	-	-	-	-	3	0.1	4.00 [2.00, 4.00]
Nasal discharge	-	-	-	106	11.3	-	-	-	106	11.3	4.5 [3.00, 4.00]
Shortness of breath	-	-	104	11.4	-	-	-	-	104	4.4	3.00 [1.00, 14.0]

## Data Availability

The raw data used to support the findings of this study can be provided by the authors on request.

## Supplementary Materials

The following supporting information can be downloaded at https://www.mdpi.com/article/10.3390/v17010125/s1, Table S1: PICO framework used to identify human MERS-CoV studies in Africa.
