# Supplementary material for: Low-Level Zoonotic Transmission of Clade C MERS-CoV in Africa: Insights from Scoping Review and Cohort Studies in Hospital and Community Settings
Source: Viruses. 2025 Jan 17;17(1):125. doi: 10.3390/v17010125 (PMC11768526; doi:10.3390/v17010125)
Supplement: Supplementary file 1 [file viruses-17-00125-s001.zip › viruses-3346845-supplementary.pdf]

SUPPLEMENTARY TABLES

Supplementary Table S1. *PICOS framework used to identify human MERS-CoV studies in Africa.*

	KEY WORD	MESH TERMS
**Population**	Humans, herders, slaughterhouse workers, abattoirs, healthcare workers, animal health workers, veterinarians	Health personnel, veterinarians, farmers, adult, child, aged
**Intervention/exposure**	MERS-CoV	MERS virus, MERS-CoV, Merbecovirus, Middle East respiratory syndrome – related coronavirus
**Context**	Africa	Africa, Africa Western, Africa Eastern, Africa Northern, Africa Southern. Ethiopia, Kenya, Djibouti, Somalia, Sudan, Eritrea, Mali, Tunisia, Morocco, Niger, Nigeria, Chad, Egypt
**Outcome**	Prevalence, morbidity, mortality, (incidence)	Prevalence, (incidence) infections, infestations, mortality, morbidity
